# Supplementary material for: SyNDI: synchronous network data integration framework
Source: BMC Bioinformatics. 2018 Nov 6;19:403. doi: 10.1186/s12859-018-2426-5 (PMC6219086; doi:10.1186/s12859-018-2426-5)
Supplement: Supplementary file 5 — Files of the example presented in Additional file 4. This additional file is a zip package that contains all files used in Additional file 4. Detailed descriptions of each of these files are presented in a README file included in this zip package. (GZ 30411 kb) [file 12859_2018_2426_MOESM5_ESM.gz › SyNDITools/Meme2Fimo/meme_4.9.0_4.tar.gz!/meme_4.9.0/doc/alphabets.html]

MEME Suite Alphabets


# MEME Suite Alphabets

- The two IUPAC alphabets are allowed.
  - The protein alphabet contains twenty
    characters for amino acids ("ACDEFGHIKLMNPQRSTVWY"),
    and is augmented by four more ambiguous characters ("BUXZ").

    ```
        A  alanine                         P  proline
        B  aspartate or asparagine         Q  glutamine
        C  cystine                         R  arginine
        D  aspartate                       S  serine
        E  glutamate                       T  threonine
        F  phenylalanine                   U  any
        G  glycine                         V  valine
        H  histidine                       W  tryptophan
        I  isoleucine                      Y  tyrosine
        K  lysine                          Z  glutamate or glutamine
        L  leucine                         X  any
        M  methionine
        N  asparagine
    ```
  - The four-character DNA alphabet ("ACGT") is augmented by eleven
    ambiguous characters:

    ```
        A --> adenosine                 M --> A C (amino)
        C --> cytidine                  S --> G C (strong)
        G --> guanine                   W --> A T (weak)
        T --> thymidine                 B --> G T C
                                           D --> G A T
        R --> G A (purine)              H --> A C T
        Y --> T C (pyrimidine)          V --> G C A
        K --> G T (keto)                N --> A G C T (any)
    ```
  - Any other characters (digits, punctuation, etc.) are
    illegal.
